# Exercise and *Phellodendron amurense* extract differentially impinge on oxidative stress response pathways in a mouse model of prostate cancer

**DOI:** 10.1038/s41598-026-42892-3

**Published:** 2026-03-05

**Authors:** Darpan I. Patel, Paul Rivas, Yidong Chen, Zhao Lai, Yuji Ikeno, Addanki P. Kumar, Rita Ghosh

**Affiliations:** 1 School of Nursing, University of Texas Medical Branch at Galveston, Galveston, TX USA; 2 School of Nursing, University of Texas Health Science Center at San Antonio, San Antonio, TX USA; 3 Mays Cancer Center, University of Texas Health Science Center at San Antonio, San Antonio, TX USA; 4 Barshop Institute for Longevity and Aging Studies, University of Texas Health Science Center at San Antonio, San Antonio, TX USA; 5 Department of Population Health Sciences, University of Texas Health Science Center at San Antonio, San Antonio, TX USA; 6 Greehey Children’s Cancer Research Institute, University of Texas Health Science Center at San Antonio, San Antonio, TX USA; 7 Department of Molecular Medicine, University of Texas Health Science Center at San Antonio, San Antonio, TX USA; 8 Department of Pathology and Laboratory Medicine, University of Texas Health Science Center at San Antonio, San Antonio, TX USA; 9 Department of Urology, Long School of Medicine, University of Texas Health Science Center at San Antonio, San Antonio, TX USA; 10 Geriatric Research and Education Center, Audie L. Murphy VA Hospital South Texas Veterans Health Care System, San Antonio, TX USA

**Keywords:** Prostate cancer, Reactive oxygen species, Oxidative stress response, Exercise, *Phellodendron amurense*, Transcriptome modulation, Cancer prevention, Urological cancer, Gene expression analysis, Urology

## Abstract

Prostate cancer is the most common non-cutaneous cancer in men in the United States. Oxidative stress, induced by chronic prostatic inflammation, impairs normal protective mechanisms in the prostate and increases the risk for cancer progression. We reported that *Phellodendron amurense* extract (Nexrutine) had similar effects as exercise in reducing highly aggressive tumors in the transgenic adenocarcinoma of the mouse prostate (TRAMP) model. Here, we examined changes in the transcriptome to understand how these two interventions affect prostate cancer. Twenty-four, 8–10 weeks old male TRAMP mice, were randomized to control, exercise, and *Phellodendron amurense* extract intervention. At the end of the study, prostate tumors were excised, weighed, and processed for immunohistochemistry and transcriptome analysis. Changes in gene expression (≥ 1.5-fold change, adjusted *p* < 0.05), gene ontology and pathway analyses were carried out. No significant difference was observed in body or genitourinary weight between groups. There was differential response to interventions with greater percentage of high-grade tumors in the control group. Triglyceride, metabolic processes and localization, transport processes were affected by exercise and *Phellodendron amurense* extract, respectively. At the transcriptome level, our results suggest that both interventions differentially affect pathways that allow them to overcome high oxidative stress threshold and protect against high-grade tumors.

## Introduction

Active lifestyle has been suggested to reduce the onset and progression of many cancers, including prostate cancer (PCa) Observational studies have shown that an increased level of physical activity is associated with decreased risk of PCa diagnosis^1,2^. Even in men diagnosed with prostate cancer, higher levels of physical activity are associated with lower risk of aggressive tumors^3^. Preclinical studies have demonstrated that exercise can suppress prostate tumor aggressiveness^4^, modulate the tumor microenvironment^5^, and alter pro-tumorigenic cytokine milieu^6^. Similarly, exercise has also been demonstrated to improve treatment response when administered as an adjuvant to traditional therapies^7^. However, due to comorbidities and/or age-related reduced physical capabilities, some men with prostate cancer may be unable to meet the physical activity guidelines that have been reported to decrease risk and improve quality of life for these patients. However, the molecular changes associated with exercise intervention in prostate cancer models is not well elucidated and is a focus of this study.

We recently identified the potential of Nexrutine to function as an exercise analog^4^. Nexrutine is a natural product derived from the bark of the cork tree, *Phellodendron amurense,* a native to Asia that belongs to the *Rutaceae* family. In traditional Chinese Medicine, Nexrutine has been historically used to treat inflammatory conditions and has also been used as an anti-bacterial agent. The major phytoconstituents of Nexrutine include berberine, palmatine, magnoflorine, jatrorrhizine, limonin and phellodendrine of which berberine and palmatine have shown greatest anti-cancer biological effects^8^. Previous work by our group has demonstrated the positive effects of *Phellodendron amurense* in suppressing tumor cell proliferation and inhibiting prostate cancer in murine models^9^. We have previously reported that Nexrutine, like exercise, reduces tumor aggressiveness, impedes inflammatory signaling and improves anti-tumor response in an adjuvant setting in murine models of prostate cancer^4^. Further, our group observed a reduced concentration of prostate specific antigen in a window of opportunity safety trial of Nexrutine given to men with prostate cancer undergoing prostatectomy or radiation therapy^10^.

Chronic prostatitis or inflammation of the prostate is a key risk factor for prostate cancer and oxidative stress is an important component in the pathogenesis of inflammation^11^. Elevated oxidative stress, a characteristic feature of PCa cells is known to play pivotal roles on prostate cancer development and progression^12^. Additionally, oxidative stress due to androgen deprivation therapy leads to adaptive survival of cancer cells underscoring the importance of oxidative stress across the disease spectrum of PCa^13^. Reports show that ability to quench reactive oxygen species (ROS) is crucial for cell survival and differentiation and the ability of cells to adapt to elevated cell intrinsic oxidative stress is involved in malignant transformation and disease progression^14–17^. While cancer cells have a higher threshold of oxidative stress tolerance, our previous work has shown that the ability of *Phellodendron amurense* to disrupt this threshold leads to cancer cell killing^18^.

Against this backdrop, the aim of this study was to determine overall changes in the transcriptome in response to exercise, and *Phellodendron amurense* extract interventions. The goal of this comparative study was to add novel molecular insights into the impact of aerobic exercise and *Phellodendron amurense* extract supplementation on making prostate tumors vulnerable to oxidative stress, thus protecting against advanced tumors.

## Results

### Exercise and *Phellodendron amurense* extract decreased tumor grade

All mice in this study, with one exception, were able to complete the study. A single mouse from the exercise group died idiopathically in week 8 of the study. At necropsy, this animal had necrotic prostate tissue, and therefore, was not included in the analysis. No significant differences were observed in genitourinary complex mass or genitourinary complex mass-to-body mass percentage at the end of the 12-week intervention across arms (Table 1). Animals in the exercise group ran 10.40 ± 3.0 km per day (range: 7.9–12.4 km/day). Body mass tended to be lower (F = 6.795; p = 0.08) in the *Phellodendron amurense* extract group compared to controls.

**Table 1:**
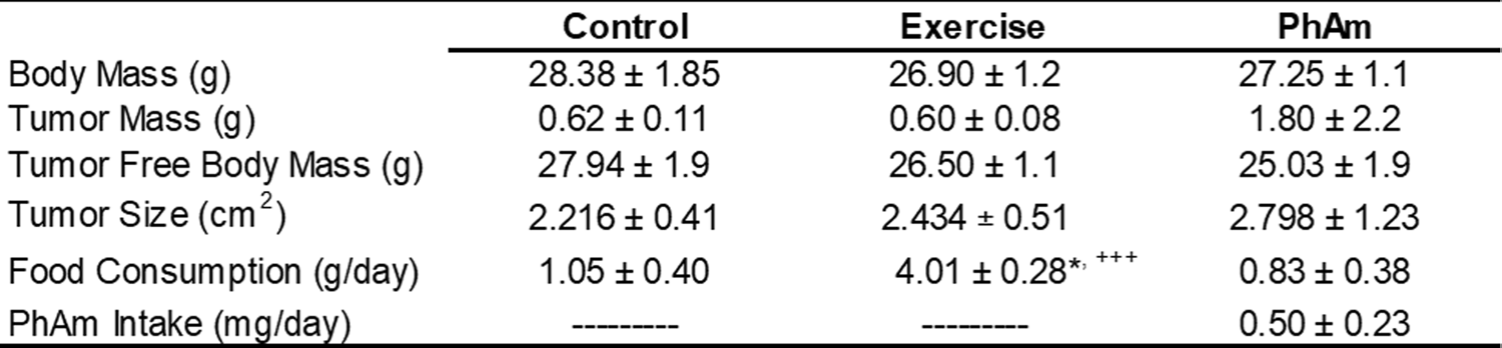
Animal characteristics at termination of study.

**Fig. 1:**
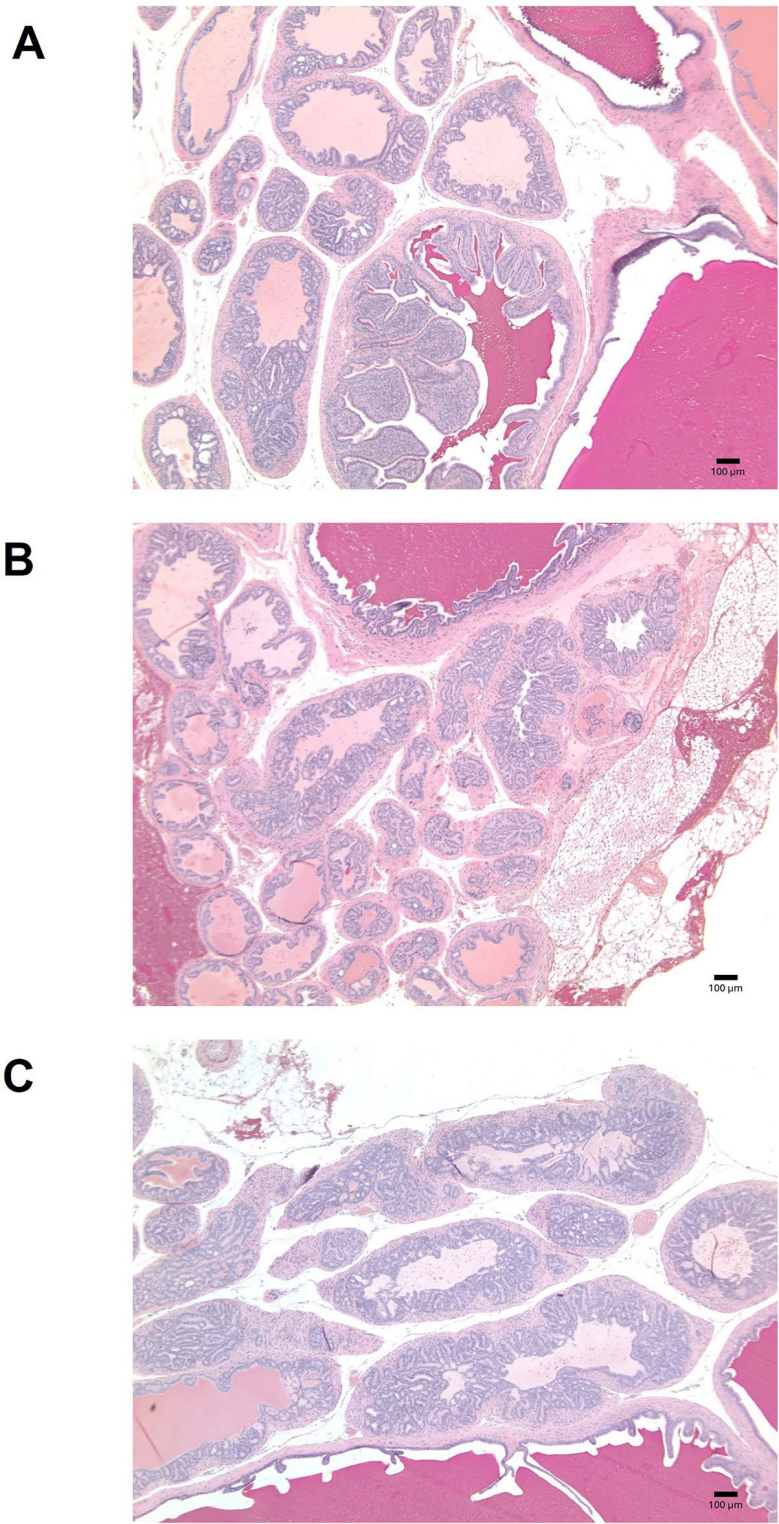
Histopathology of tumors from TRAMP mice imaged at 40x. Tumors were excised from all the animals at the time of study termination and stained with Hematoxylin and Eosin. Representative images are shown with pathology score indicative of the majority of mice in the group. **A**. H&E from an animal in the control group with a pathology score of 4 (without intervention). **B**. H&E of a representative tumor from an animal in the exercise intervention group with a pathology score of 4. **C**. H&E from an animal fed with p*hellodendron amurense* containing diet with a pathology score of 4.

**Fig. 2:**
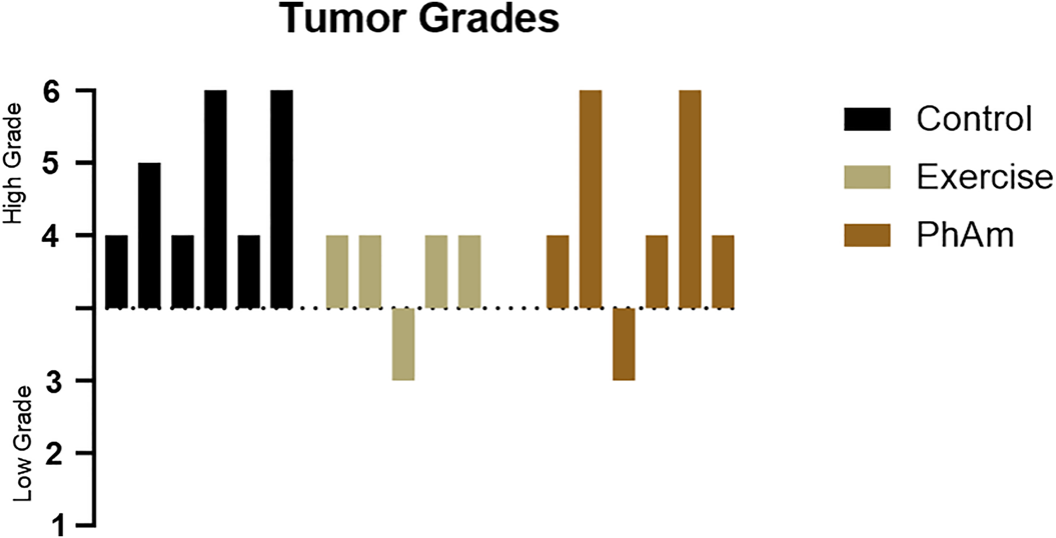
Waterfall diagram presenting pathology scores of extracted tumors.

No significant differences were observed between groups with respect to tumor pathology scores (Fig. 1). However, 50% of mice in the control group exhibited moderately or poorly differentiated tumors (grade 5 or 6). In contrast, 4 out of 5 mice in the exercise only group showed well differentiated tumors (grade 4) including prostatic intraepithelial neoplasia (PIN, grade 3) lesions in one animal (Fig. 2). In addition, 3 out of 6 animals from the PhAm group showed well differentiated (grade 4) while 1 of the 6 animals exhibited PIN lesions (Fig. 2). In summary, tumor pathology ranged from moderate to poorly differentiated phenotype in 50% of mice from control group. On the other hand, none of the mice from the exercise and 33% from PhAm group showed poorly differentiated tumors. However, despite differences in pathology, tumor mass and tumor size were higher in animals from PhAm group relative to control or exercise group of animals (Table 1). Although the precise reason for this is unclear at this time, we believe that fatty tissue surrounding the tumor could have contributed to this. In addition, animals in the PhAm group may have had unequal access to PhAm because it was provided in the diet. Given the group housing of animals all animals in a given cage may not have consumed equal amounts of food.

### Differential tumor transcriptional signature in response to aerobic exercise and *Phellodendron amurense* extract interventions 

**Fig. 3:**
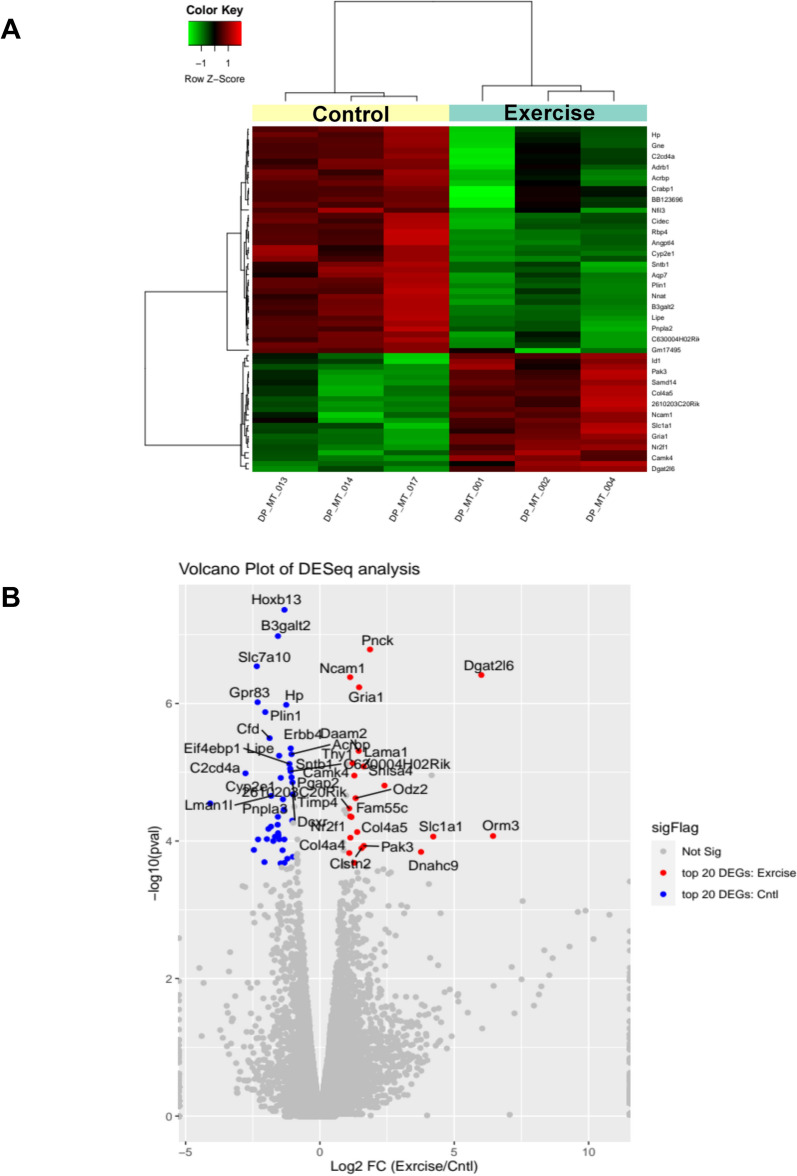

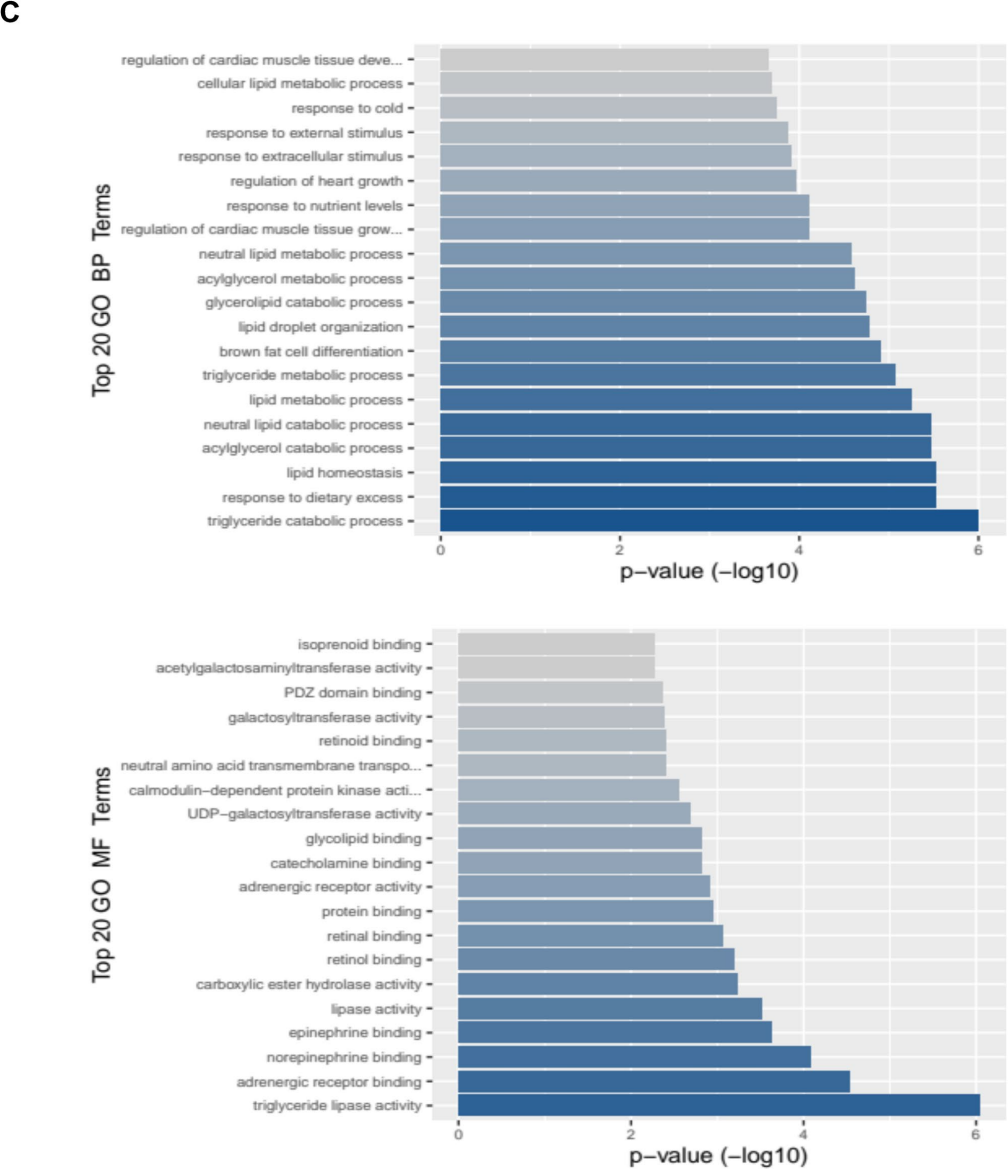

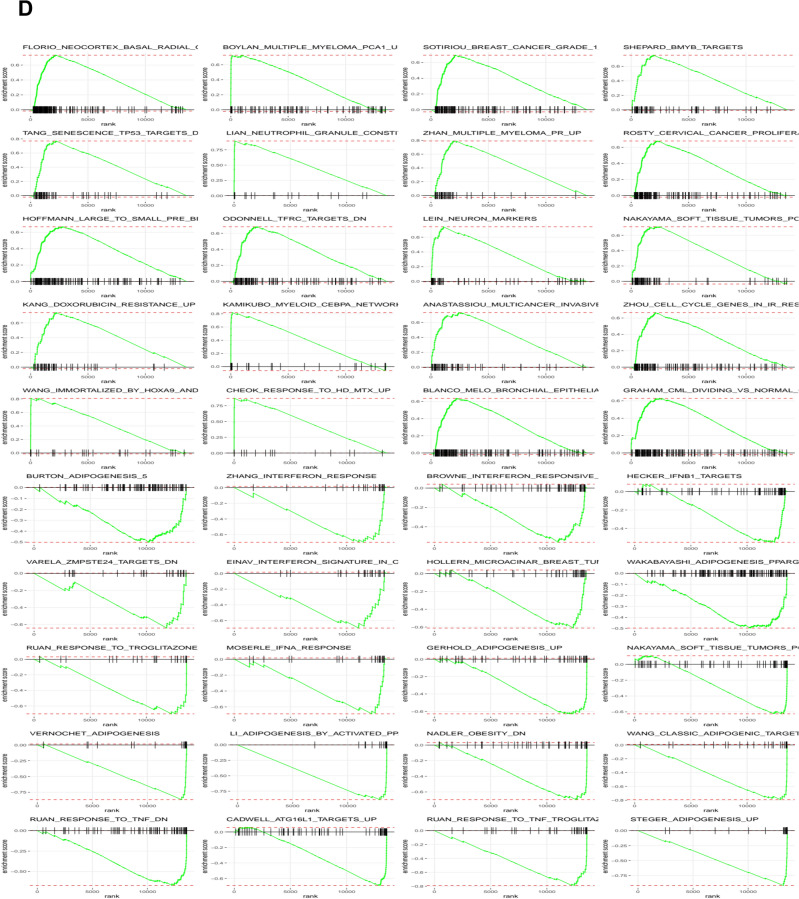
Transcriptomic changes associated with exercise intervention in TRAMP mice. **A**. Heat map of transcriptomic changes between control TRAMP mice that did not receive any intervention and animals that were subject to voluntary wheel running. **B**. Volcano plots of the differentially expressed genes (DEGs) in the exercise group compared with the no exercise control animals. **C.** The top 20 biological processes and molecular functions associated with voluntary wheel running. **D**. Gene set enrichment of the identified DEGs.

To investigate the molecular mechanisms by which aerobic exercise can inhibit progression of prostate tumors to aggressive stages, we subjected representative prostate tumors to transcriptomic analysis (n = 3 per group). By comparing the transcriptome of the exercising group to the control mice, we identified 32 differentially expressed genes (DEGs) that were significantly modified (adj p < 0.05) and demonstrated ± 1.5-fold change. Among the top 20 transcripts that decreased in the exercise group were genes involved in lipolysis such as *Lipe*, *Plin1*, *Slc7A10* (Table 2). Among the top transcripts with higher expression in the exercise group was *Dgat2L6* that is involved in the synthesis of diglycerols and triglycerols. Gene ontology analysis of the DEGs that were increased in the exercise group revealed enrichment of biological processes associated with triglyceride metabolic processes and lipid metabolic processes (Fig. 3A). The molecular functions that were most impacted by exercise included triglyceride lipase activity, adrenergic receptor binding, norepinephrine and epinephrine binding, and lipase activity (Fig. 3B). Figure 3C,D present impacted biological pathways, molecular function and gene set enrichment data.

**Table 2:**
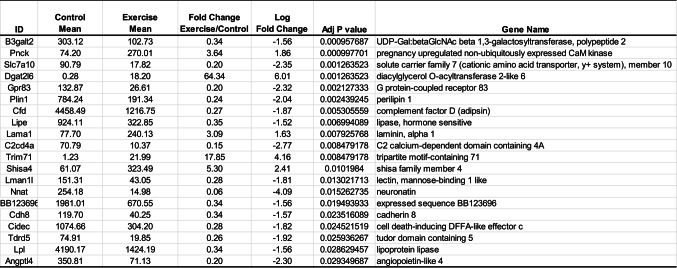
Top 20 differentilly expressed genes in the execise group compared to control.

**Fig. 4:**
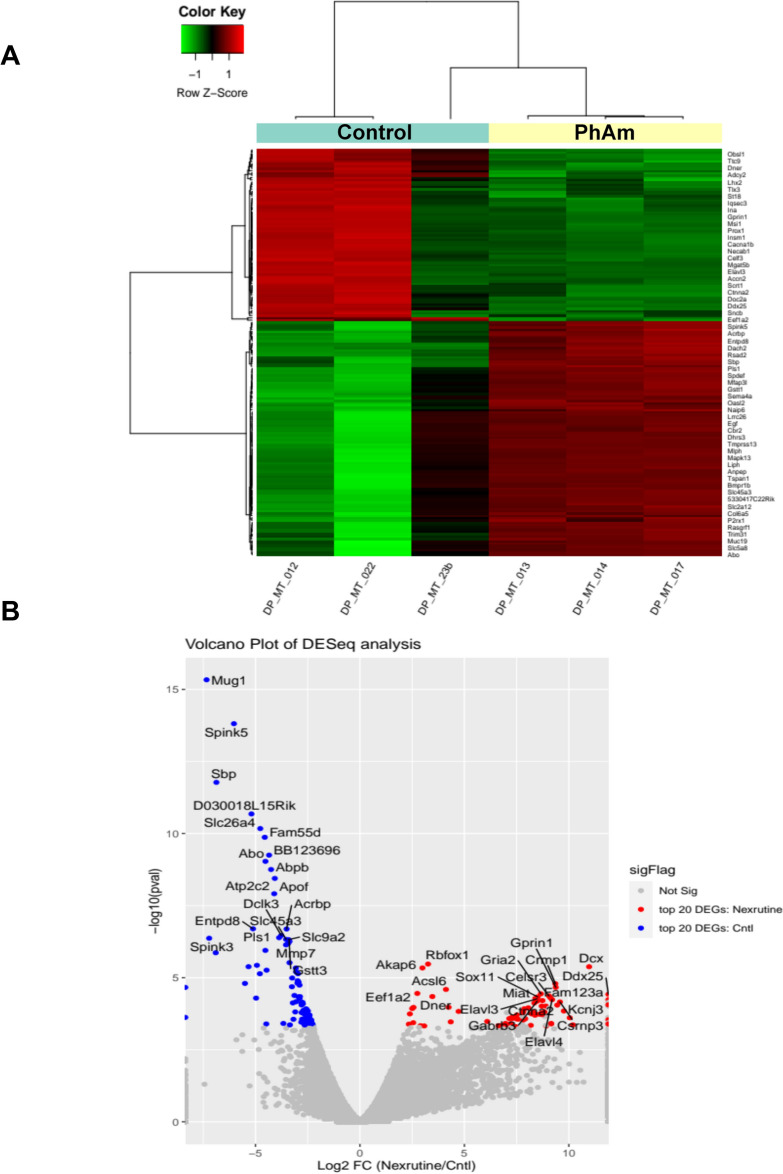

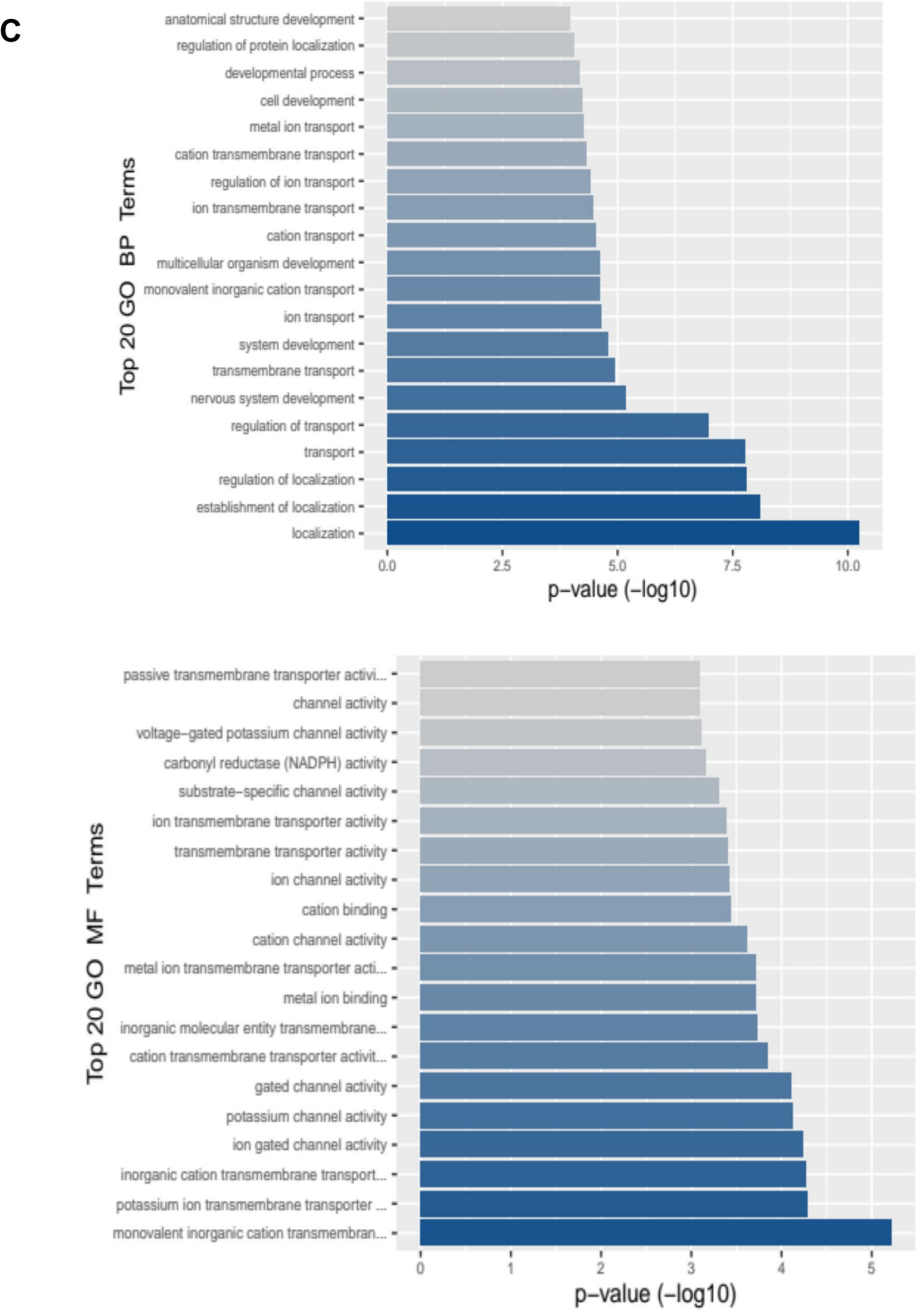

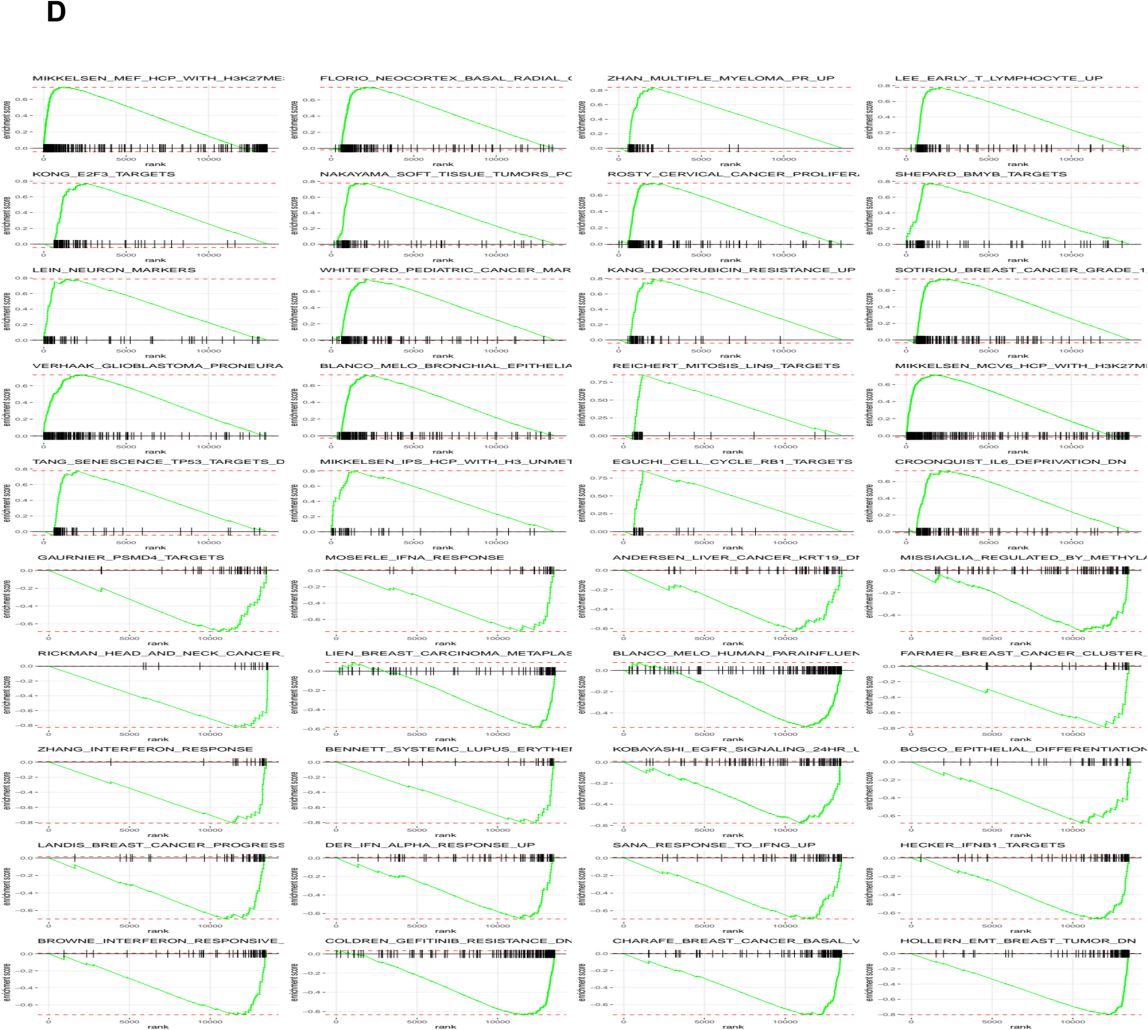
Transcriptomic changes associated with *Phellodendron amurense* intervention in TRAMP mice. **A**. Heat map of transcriptomic changes between TRAMP mice fed with control diet and animals fed a diet with *Phellodendron amurense* **B**. Volcano plots of the differentially expressed genes (DEGs) in the *Phellodendron amurense* fed group compared with control animals with normal diet. **C.** The top 20 biological processes and molecular functions associated with consumption of *Phellodendron amurense*. **D**. Gene set enrichment of the identified DEGs.

In comparison to control samples, *Phellodendron amurense* extract was found to alter the expression of 176 transcripts in prostate tumor tissue (adj p < 0.05 with a ± 1.5-fold change). Of these, 100 were significantly decreased and 76 were significantly increased. Among the significantly decreased transcripts, we found serine protease inhibitors (*Spink3, Spink5*) and solute carrier family proteins (*Slc26a4, Slc45a3, Slc5a8, Slc9a2, Slc10a5, Slc2a12, Slc44a3, Slc7a4*). Among the increased transcripts were neuronal factors, such as *Dcx* and *Sncb* (Table 3). Gene ontology identified major effects on localization (i.e. spatial distribution of the RNA), both the establishment and regulation of localization, as well as transport pathways, specifically regulation of transport, transmembrane transport, and ion transport (Fig. 4A). Transcriptomic data also showed that functions associated with inorganic cation transmembrane transport, potassium ion transmembrane transport as well as potassium channel and ion gated channel activity increased significantly in the *Phellodendron amurense* extract group compared with animals in the control arm (Fig. 4B). Figure 4C,D present impacted biological pathways, molecular function and gene set enrichment data.

**Table 3:**
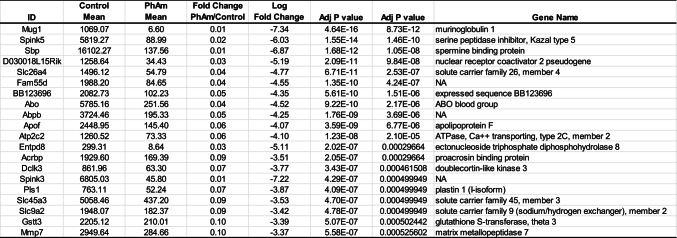
Top 20 differentially expressed genes in the diet *Phellodendron amurense* fed group compared to controls.

In evaluating the overlap between modified DEGs, we observed only 2 that were similar between the two group (*Bb123696*; and *Lman1l*), both of which had increased expression in response to both interventions.

## Discussion

Exercise has been suggested as an intervention to prevent prostate cancer. During exercise, ROS is mainly produced by the mitochondrial respiratory chain and the amount of ROS produced has been linked to positive or negative effects especially in the skeletal muscle system which is the most relevant to exercise^19–23^. Normally cells are equipped with endogenous mechanisms to neutralize ROS. However, in cancer cells the combination of higher threshold of ROS due to higher metabolism and frequent loss or malfunction of endogenous antioxidant enzymes results in greater accumulation of ROS. The lipid rich nature of cell membranes makes them highly susceptible to ROS damage due to lipid oxidation. In skeletal muscles, ROS has been shown to damage the polyunsaturated fatty acids in the membrane lipid bilayer and produce unsaturated fatty acid free radicals that ultimately leads to the formation of lipid peroxide. In addition to phospholipid damage, ROS also damages membrane proteins causing crosslinks between lipids and proteins or proteins to proteins which alters membrane integrity, fluidity, diminishing membrane receptor and membrane enzymatic activity^24^. The high energy demands of cancer cells due to uncontrolled proliferation requires rapid production of macromolecule building blocks^25^. As such, lipids are also considered a building block in addition to nucleic acids and proteins. In this context, lipid metabolism is intensely affected in rapidly proliferating cancer cells due to increased demand for fatty acids that have to be incorporated into membrane phospholipids^26^. Lipids regulate plasma membrane fluidity, membrane remodeling and intracellular signaling, processes that serve as important players during tumorigenesis and cancer cell metastasis^27–29^. Fatty acid catabolism as a source of mitochondrial energy production is accompanied by ROS which allows cancer cells to adapt to avoid ROS associated toxicity by upregulating anti-ROS defense mechanisms. Fatty acid oxidation during exercise requires lipolysis (hydrolysis of triacylglycerols). Subjecting animals to voluntary aerobic exercise identified significant changes in processes involved in triglyceride, acylglycerol, neutral lipid, glycerolipid, and lipid droplet organization. *Lipe*, was significantly downregulated in the exercise arm compared with controls. *Lipe* catalyzes the hydrolysis of triacylglycerols and fatty acid esters^30,31^. We also found significant decrease in *Plin1* and *Gpr83* transcripts in the tumors of exercising animals. *Plin1*coats lipid droplets and protects them from the action of lipases such as *Lipe* which produce the free fatty acids and glycerol to feed lipid metabolism^32^. *Gpr83* is an orphan G protein-coupled receptor that is implicated in the peripheral generation of Foxp3^+^Tregulatory cells and in body weight regulation^33^. Exercise significantly upregulated *Dgat2l6* expression. *Dgat2l6*is involved in the synthesis of acylglycerols^34^. These results suggest that disruption of lipid metabolism in the exercise group may potentially attenuate associated ROS generation and manifest in decreased tumor differentiation stage.

In the animals fed *Phellodendron amurense* extract containing diet, we found several significantly decreased transcripts associated with oxidative stress related to membrane lipids, proteins, and tumor growth. Functions of membrane proteins such as ion channels, transporters and receptors can be disrupted by cholesterol present in cellular membranes. Modulation of the activity of membrane systems is an oxidative stress response and the activity can either be stimulated or blocked during oxidative stress^35^. Our results show that *Phellodendron amurense* extract intervention inhibited transcripts of members of the solute carrier superfamily of proteins such as *Slc26a4*, *Slc45a3* and *Alc9a2*, the ATP driven pump (*Atp2c2*) that supplies Ca^2+^ and Mn^2+^ ions at the Golgi membrane. Solute carrier proteins serve as gatekeepers and control the transmembrane flux of inorganic ions, sugars, amino acids, nucleotides, fatty acids, neurotransmitters, and drugs. *Slc26a4* like other members of this family has versatile substrate selectivity, generally accepting divalent, monovalent anions, exchanging chloride for inorganic anions such as bicarbonate, hydroxyl, sulphate and iodide, or small organic anions such as formate, oxalate and operating in Cl^−^/HCO_3_^−^ exchange mode^36^. Atp2c2 facilitates Ca^2+^ influx channels at the plasma membrane and active Ca^2+^ transport at the Golgi apparatus^37^. The ability of *Phellodendron amurense* extract to significantly decrease *ApoF*, *Pls1*, *Mmp7* transcripts suggests that the extract has protective effects against prostate cancer. *ApoF* is involved in cholesterol transfer between lipoproteins, that is thought to contribute to cholesterol accumulation in lipid droplets and is increased in prostate cancer^38^. L-plastin is an actin cross-linking protein regulated by steroid hormones that is involved in filopodia formation that play important roles in cell motility, a key metastatic feature of cancer cells^39–41^. MMP-7, a member of the matrix metalloproteinase family, is involved in the disintegration of the extracellular matrix including proteoglycans, fibronectin, elastin, and casein, promoting osteolysis and is associated with worse prostate cancer survival^42,43^. ROS is generally known to be an activator of MMPs due to their ability to oxidize cysteine thiol groups of the pro-domain during inflammation, the latter being commonly seen in prostate cancer^44^. These decreased transcripts suggest that *Phellodendron amurense* extract may be able to protect against cancer progression to metastatic disease by inhibiting a combination of extracellular matrix degradation, development of filopodia and changes in membrane fluidity that facilitate cancer cell motility. Our work also provides evidence that *Phellodendron amurense* extract can lower transcripts that are associated with high oxidative stress that is used as an adaptive mechanism by cancer cells for survival. For example, *Phellodendron amurense* extract intervention decreased the expression of *Dclk3* which is involved in modulating levels of iron and ROS and *Gstt3* which is involved in neutralizing electrophilic and hydrophobic compounds. These results are in keeping with our previous work that showed the ability of *Phellodendron amurense* to disrupt the higher threshold of oxidative stress in melanoma cells, needed for their survival. Elevated oxidative stress, a characteristic feature of prostate cancer cells is known to play pivotal roles on prostate cancer development and progression^12^. Additionally, oxidative stress due to androgen deprivation therapy leads to adaptive survival of cancer cells underscoring the importance of oxidative stress across the disease spectrum of prostate cancer^13^. Reports show that ROS production and depletion is crucial for cell survival and differentiation and adaptation to elevated cell intrinsic oxidative stress is involved in malignant transformation and disease progression^14–17^.

The transcriptomic data presented here suggest that both exercise and *Phellodendron amurense* extract are beneficial interventions against prostate cancer albeit through their effects on different pathways. Theoretically, this raises a number of options for future research, such as combination treatments, dose escalation studies of PhAm, and potentially the role of PhAm in modifying body composition (thus decreasing risk of prostate cancer). Practically, these data offer hope to incapacitated prostate cancer patients that may be unable to resort to aerobic exercise to prevent prostate cancer progression. Interestingly, the changes in transcriptomic data reflected better on the tumor grade of animals in the exercise group compared with the *Phellodendron amurense* extract fed group of animals. It is possible that since every animal had access to its own running wheel in its cage, the beneficial effect was reflected better in this group. Contrastingly, the animals in the *Phellodendron amurense* extract fed group may have consumed different amounts of the diet which could therefore affect tumor grade differentially in this group. Further studies that measure clear-cut *Phellodendron amurense* extract consumption may be needed for a head-to-head comparison of the benefits of the two interventions against prostate cancer. Additionally, the small sample size used for transcriptomic evaluation is a limitation of our ability to uncover generalizable effects of our interventions. Nonetheless, this study shows the potential benefits of *Phellodendron amurense* extract intervention in modulating transcripts that are associated with oxidative stress response and prostate cancer progression in this aggressive preclinical model of prostate cancer.

## Materials and methods

### Transgenic mouse model

Transgenic adenocarcinoma of the mouse prostate (TRAMP) mice in C57BL/6 background obtained from Jackson Laboratories (The Jackson Laboratories, Bar Harbor, Maine) were used in this study. TRAMP mice were developed by prostate-specific expression of SV40 large T antigen using the rat probasin promoter^45^. TRAMP mice develop prostate tumors with 100% frequency, in progressive stages that facilitates preclinical studies in the prevention, intervention and regression setting as demonstrated by our group and others^9,46–48^.

### Experimental design

**Fig. 5:**
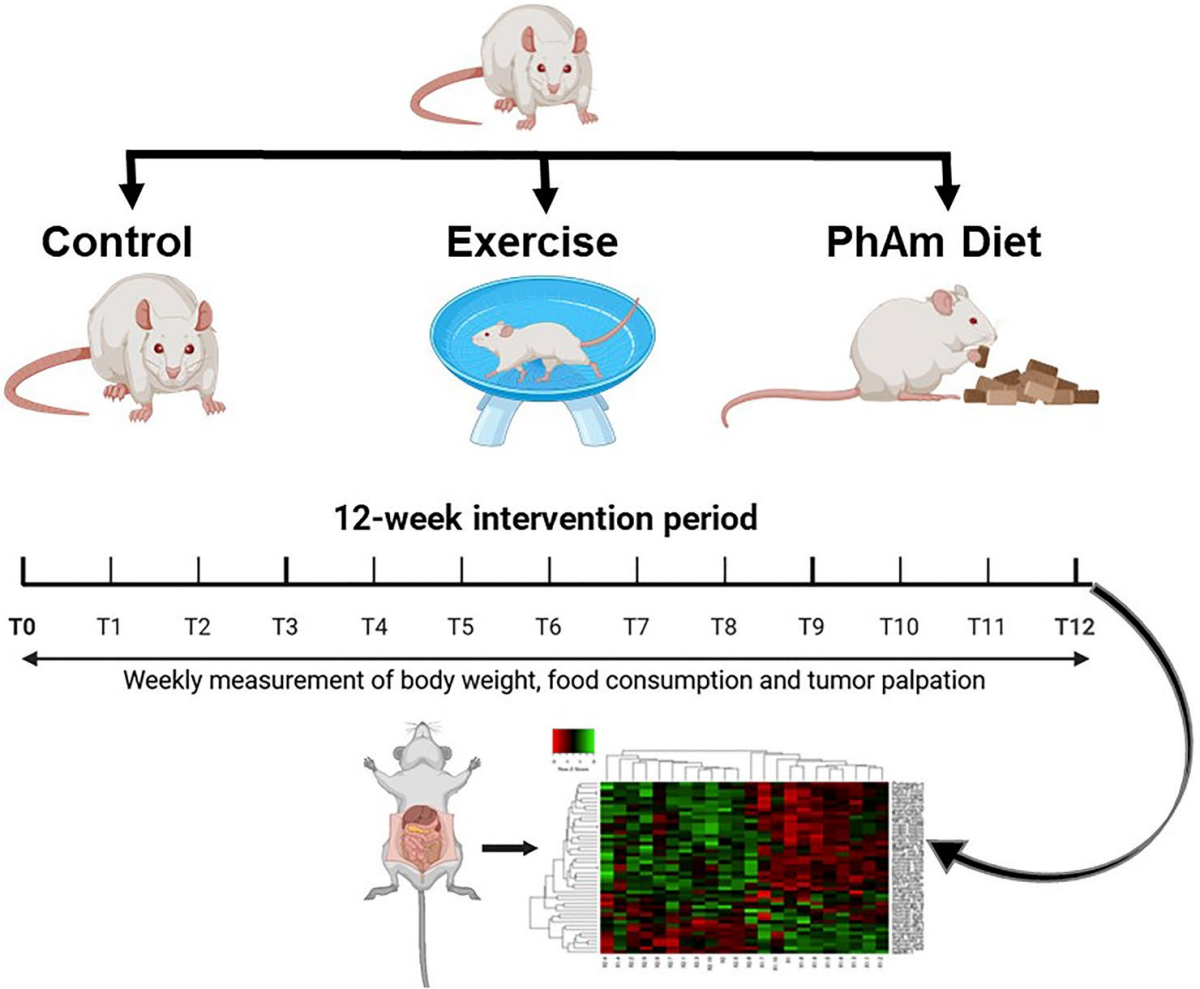
Study design. Eighteen mice were equally randomized to either the control group, exercise or *Phellodendron amurense* (PhAm) group. After the 12-week intervention, tumors were collected for pathology and transcriptomic analysis.

Animal care and handling was maintained in accordance with federal guidelines. This study was approved by the Institutional Animal Care and Use Committee at the University of Texas Health Science Center at San Antonio and comply with the ARRIVE guidelines. Eighteen male (TRAMP; in C57BL/6 background) were used in this study. Mice at 8–10 week of age were randomized to one of three treatment groups: control, exercise, *Phellodendron amurense* extract (n = 6 each). Mice were maintained in a climate-controlled environment with a 12-h light–dark cycle with diet and water provided ad libitum*. Phellodendron amurense* extract was supplied in the diet and running wheel was used as the exercise intervention. The control group mice were group housed had access to AIN-93 diet ad libitum and did not have access to running wheel. Body weight changes, food consumption and distance run on the running wheel were measured weekly for the 12-week intervention. Animals were euthanized at the end of intervention with inhaled CO_2_ with exsanguination as a secondary method to confirm death. After confirmation, the prostate-seminal vesicle complex was excised and weighed. Tumor size was measured using ImageJ (version 1.54 g, Java 1.8.0_345, Wayen Rasband, U.S. National Institutes of Health, Bethesda, MD, USA; website: http://imagej.org). Part of the tumor was prepped for histopathological analysis and part was flash frozen for RNA extraction and transcriptomic analyses. The study design is presented in Fig. 5.

### Voluntary wheel running

Mice randomized to the exercise groupwere singularly housed and given access to a 15.5 cm diameter low-profile running wheel (Med Associates, Inc., Georgia, VT). Running wheels were placed in the cage of each mouse. Wheel revolutions were recorded wirelessly on a computerized activity monitoring systerm. This modality reflects activity that is consistent with exercise behavior; is inversely correlated with mortality and occurs within the routine diurnal rhythm patterns of mice, providing an exercise stimulus in a stress-free environment. This modality is consistant with previous research demonstrating exercise efficacy in suppressing tumor aggressiveness^4^. Mice were housed individually to ensure accurate measurement of physical activity.

### Preparation of *Phellodendron amurense* extract diet

*Phellodendron amurense* extract was purchased from Chinese Herbs Direct (Torrance, CA, USA) and tested for purity and bioequivelance to our previous studies (data not shown). We used 600 mg/kg *Phellodendron amurense* extract based on results from previously published studies^9^. Diet pellets of *Phellodendron amurense* extract in AIN-93 was prepared at Dyets, Inc. (Bethlehem, PA, USA). Mice randomized to the *Phellodendron amurense* extract were group housed and provided this specialized diet ad libitum. Food consumption was assessed weekly.

### Tumor histology

Following euthanasia, tumors were excised, weighed and a part of the tumor was fixed in 10% neutral buffered formalin. Formalin fixed tumors were embedded in paraffin and sections were cut, placed on slides and stained with hematoxylin and eosin for histopathological assessment. Prostate lesions were scored using grading ranges established previously by our group^9^. Histopathological grades range from 1–6 with (1) being normal, non-cancerous tissue, (2) low grade PIN, (3) high grade PIN, (4) well-differentiated cancerous lesions, (5) moderately differentiated cancerous lesions and (6) poorly-differentiated cancerous lesions. Three sections from each tumor were analyzed for histopathological grade. Liver, kidney and bladder were also evaluated for metastesis.

### RNA-seq library preparation and next generation sequencing

Tumor tissue from 3 animals from each group was used for this portion of the study. TRIzol (Thermo Fisher) was used to extract total RNA from the tumors. The quality of total RNA was checked by Agilent Fragment Analyzer (Agilent Technologies, Santa Clara, CA), and RNA integrity scores were determined and RNA with a score of > 5 was used for RNA-sequencing. Approximately 500 ng total RNA was used for RNA-seq library preparation by following the KAPA Stranded RNA-Seq Kit with RiboErase (HMR) sample preparation guide (Cat. No: KR1151, KAPA Biosystems, USA) as previously described by our group^49,50^. First step in the workflow was the depletion of rRNA by hybridization of complementary DNA oligonucleotides. This was followed by treatment with RNase H and DNase to remove rRNA duplexed to DNA and original DNA oligonucleotides, respectively. After rRNA removal, the mRNA was fragmented into small pieces using divalent cations under elevated temperature and magnesium. The cleaved RNA fragments were copied into first strand cDNA using reverse transcriptase and random primers followed by second strand cDNA synthesis using DNA Polymerase I and RNase H. Strand specificity was achieved by replacing dTTP with dUTP in the Second Strand Marking Mix (SMM). These cDNA fragments then went through an end repair process, the addition of a single ‘A’ base, and then ligation of the adapters. Products were then purified and enriched with PCR to create the final RNA-Seq library. RNA-Seq libraries were subjected to quantification process by the combination of Qubit and Bioanalyzer, pooled for cBot amplification and subsequently sequenced with 50 bp single read sequencing run with Illumina HiSeq 3000 platform. After the sequencing run, demultiplexing with Bcl2fastq2 was employed to generate the fastq file for each sample. An average of 25-30M reads were obtained for this set of samples.

### Data analysis

We used Genome build (University of California at Santa Cruz genome browser) and TopHat for genome alignment. Reads for each sample was counted by HTseq and the R package DESeq was used to normalize data and find group-pairwise differential gene expression. We used three criteria to identify differentially expressed genes; at least one sample type had an average reads per kilobase of transcript per million mapped reads (RPKM) > 1, false discovery rate (FDR) adjusted *p*-value < 0.05 and fold change > 1.5 (either way).

One-way analysis for between group comparisons for all end of study variables was conducted using Kruskal–Wallis test. Between group comparisons to evaluate pre-post changes was performed using paired t-test. Significance was set at p < 0.05 and data are presented at mean ± standard deviation.

## Data Availability

The datasets generated and/or analyzed during the current study are available in the GEO repository [accession number GSE293221, available at https://www.ncbi.nlm.nih.gov/geo/query/acc.cgi?acc=GSE293221].
